# The mammary glands of cows abundantly display receptors for circulating avian H5 viruses

**DOI:** 10.1128/jvi.01052-24

**Published:** 2024-10-10

**Authors:** María Ríos Carrasco, Andrea Gröne, Judith M. A. van den Brand, Robert P. de Vries

**Affiliations:** 1 Department of Chemical Biology and Drug Discovery, Utrecht Institute for Pharmaceutical Sciences, Utrecht University, Utrecht, the Netherlands; 2 Division of Pathology, Department of Biomolecular Health Sciences, Faculty of Veterinary Medicine, Utrecht University, Utrecht, the Netherlands; St. Jude Children's Research Hospital, Memphis, Tennessee, USA

**Keywords:** avian influenza, H5N1, clade 2.3.4.4b, dairy cattle, mammary gland

## Abstract

Influenza A viruses (IAVs) from the H5N1 2.3.4.4b clade are circulating in dairy farms in the USA.; ruminants were presumed not to be hosts for IAVs. Previously, IAV-positive mammalian species were hunters and scavengers, possibly getting infected while feeding on infected birds. It is now recognized that H5N1 viruses that circulate in US dairy cattle transmit through a mammary gland route, in contrast to transmission by aerosols via the respiratory tract. The sialome in the cow mammary and respiratory tract is so far solely defined using plant lectins. Here, we used recombinant HA proteins representing current circulating and classical H5 viruses to determine the distribution of IAV receptors in the respiratory and mammary tract tissues of cows. We complemented our study by mapping the glycan distribution of the upper and lower respiratory tracts of horses and pigs. Most of the sialome of the cow respiratory tract is lined with sialic acid modifications, such as N-glycolyl and O-acetyl, which are not bound by IAV. Interestingly, the H5 protein representing the cow isolates is bound significantly in the mammary gland, whereas classical H5 proteins failed to do so. Furthermore, whereas the 9-O-acetyl modification is prominent in all tissues tested, the 5-N-glycolyl modification is not, resulting in the display of receptors for avian IAV hemagglutinins. This could explain the high levels of virus found in these tissues and milk, adding supporting data to this virus transmission route.

**IMPORTANCE:**

H5N1 influenza viruses, which usually affect birds, have been found on dairy farms in the USA. Surprisingly, these viruses are spreading among dairy cows, and there is a possibility that they do not spread through the air but through their milk glands. To understand this better, we studied how the virus attaches to tissues in the cow’s respiratory tract and mammary glands using specific viral proteins. We found that the cow-associated virus binds strongly to the mammary glands, unlike older versions infecting birds. This might explain why the virus is found in cow’s milk, suggesting a new way the virus could be spreading.

## INTRODUCTION

Since late 2020, we have been experiencing an unprecedented global outbreak of highly pathogenic H5Nx influenza A viruses (IAVs) (1, 2). These viruses circulate year-round from the Northern to Southern hemisphere and cause incredibly high mortality in avian species with significant transmission to mammals (3–5). The transmission route to mammals is under debate as most infected mammals are hunters and scavengers, possibly getting infected while consuming bird remains (6–9). Seal infections, on the other hand, have been known to occur for decades due to their proximity to wild waterfowl (10); however, the spread into pinnipeds of the southern hemisphere was unprecedented. The appearance of the viruses in the Antarctic and the current zoonotic event to ruminants add even more novelties to this remarkable outbreak (11). The latest isolation of IAVs in cows was an unexpected transmission event as these species were believed not to be hosts of IAVs, while they are hosts for influenza D viruses (IDVs) (12, 13). However, it quickly became apparent that mammary tract tissues are probably essential in the transmission route, as milk samples most frequently contain high viral titers (13, 14).

The molecular determinant of zoonotic capabilities of the currently widespread H5Nx viruses is currently unknown. While the E627K mutation in the polymerase is commonly found in viruses isolated from mammalian species, whether viruses with this mutation circulate in birds or if this mutation is immediately selected in mammals is under debate. Only the human isolates associated with the current cow outbreak contain this mutation (15). The HA gene has been remarkably stable with no mutations in the receptor binding site (RBS) in the 2.3.4.4b virus for years. Genotypes such as 2.3.4.4a,c, and e harbor some RBS mutations; however, these clades are minority species. Thus, it is likely that 2.3.4.4b virus HA proteins already have an optimal receptor-binding specificity, allowing their panzootic nature.

Most IAVs use sialic acids (Sias) to enter a host cell. The glycome that presents these Sias in the respiratory tract of farm animals, such as goats and cows, is poorly defined (16, 17) in contrast to several other bovine proteins, such as submaxillary gland mucins and fetuin (18–21). It is, however, known that ruminants display a set of modified Sias that other IAV host species do not. These include the N-glycolyl modification at the C5 position (5-N-glycolyl, Neu5Gc), which is created by the cytidine monophospho-N-acetylneuraminic acid hydroxylase (CMAH) (22). CMAH is a mammalian-specific enzyme that is non-functional in various influenza hosts, including humans, ferrets, dogs, and seals (23). Cows and goats express a functional enzyme and thus abundantly display Neu5Gc (24), which is not a receptor for most influenza A viruses (25). The other abundant Sia modifications in the cow respiratory tract are O-acetyls, which can be present in the 4, 7, 8, and 9 positions. The latter is the essential receptor for influenza C, D (26), and a variety of coronaviruses (27, 28) but a non-ligand for influenza A virus (29–31). The current dogma is that these Sia modifications, not present in the avian reservoir, are decoy or blocking moieties for IAVs.

To determine if 2.3.4.4b H5 influenza A viruses can bind to available receptors, we used formalin-fixed and paraffin-embedded (FFPE) tracheal, lung, and mammary gland tissues of cows and respiratory tract of horses and pigs (32). These animals were not previously infected with the HPAIV H5N1 virus. We observed that while the mammary gland of cows displays receptors for currently circulating 2.3.4.4b H5 viruses, their respiratory tract does not, confirming this transmission route. We also report that plant lectin staining does not necessarily colocalize with IAV receptors.

## RESULTS

### 2.3.4.4b viruses isolated from dairy cows have a conserved receptor binding site

**Fig 1:**
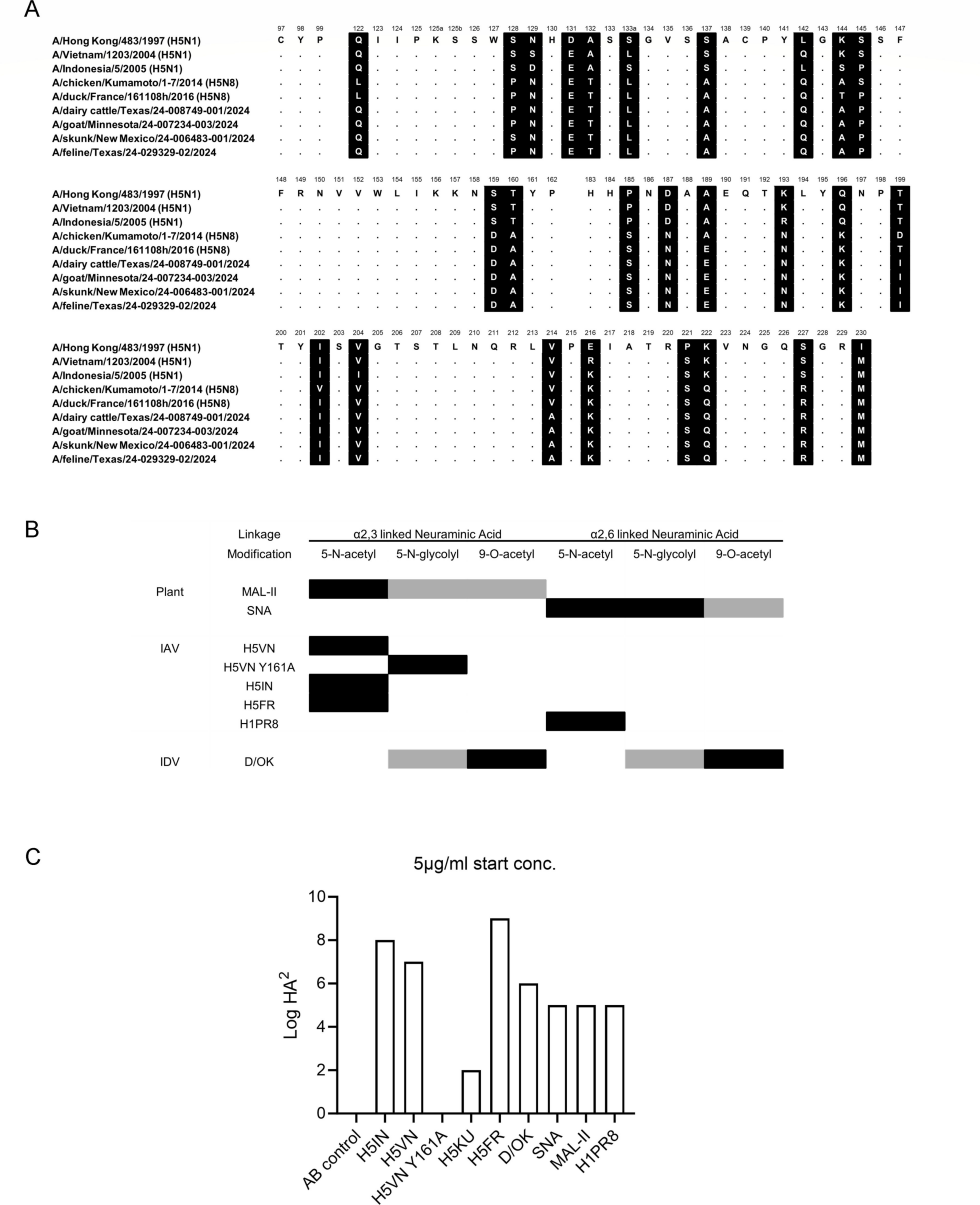
(**A**) HA receptor binding site amino acid alignment of the H5 hemagglutinins used in this study. Alignment of the receptor binding site residues with amino acid positions (H3 numbering) indicated above the alignment, non-conserved residues are highlighted in black, and dots indicate identical amino acids. Several mammalian sequence isolates in North America are shown to demonstrate the close relationship. (**B**) Overview of lectin specificity to differentially linked unmodified and modified SIA. Black boxes indicate core specificities, and gray boxes indicate possible ligands. (**C**) Hemagglutination assay with chicken erythrocytes with the lectins used for tissue stain.

The receptor binding site, the HA of IAVs, is composed of the 190 helices (AA180-195), the 130-(AA130-140), and 220-(AA220-230) loops. Since the introduction of the 2.3.4.4b clade a decade ago, the receptor binding domain has remained relatively conserved (Fig. 1A). Significant changes in 2.3.4.4 viruses, compared to the classical A/Vietnam/1203/2004 (H5VN) and A/Indonesia/05/2005 (H5IN), include the 130 loop, the loss of a glycosylation site at position 158, and significant perturbations in the 190 helix and the 222 and 227 positions in the 220 loop that are directly involved in receptor binding (33, 34). Taking A/duck/France/161108h/2016 (H5FR) as a reference, we only observe minimal amino acid changes in the recent North American mammalian-derived viruses. The mutations observed are outside the canonical RBS, namely L122Q, T144A, T199I, and V214A (H3 numbering). Of note, some cow sequences isolated later now contain A160T and thus restore the glycosylation site at position 158. Conclusively, although the 2.3.4.4b H5 viruses have been circulating for several years around the globe in different hosts, the receptor binding domain remained conserved.

### Contemporary 2.3.4.4b HA proteins bind efficiently to α2,3-linked Neu5Ac containing sialosides in mammary tract tissues of cows

A central observation of IAV-infected dairy cows is mammary gland infections (14, 17, 35). To confirm that this gland has receptors for H5 IAVs to support active replication, we used both classical and contemporary H5 proteins for tissue binding studies. We used the classical H5 derived from A/Indonesia/05/2005 (H5IN), A/Vietnam/1203/2004 (H5VN), and the Y161A (H5VN Y161A) mutant that confers binding to Neu5Gc (36). To detect 9-O-acetylated structures, we employed an enzymatically inactive influenza D virus hemagglutinin esterase fusion protein (D/OK) (12). We also used commonly employed plant lectins SNA and MAL-II (biotinylated, Vector laboratories) to determine the distribution of α2,3- and α2,6-linked Sias. To detect IAV human type receptors α2,6-linked Sias, we employed a human H1 HA derived from A/Puerto Rico/8/34 (H1PR8) (37). Lectin and HA glycan specificities are summarized in Fig. 1B.

We confirmed the biological activity of all the lectins using a traditional hemagglutination assay in which the proteins that bind erythrocytes form a mesh. All proteins can hemagglutinate chicken erythrocytes (Fig. 1C), except for the H5VN Y161A mutant, as there is no Neu5Gc in avian species. H5KU also binds less avidly than the other H5 proteins but is biologically active.

**Fig 2:**
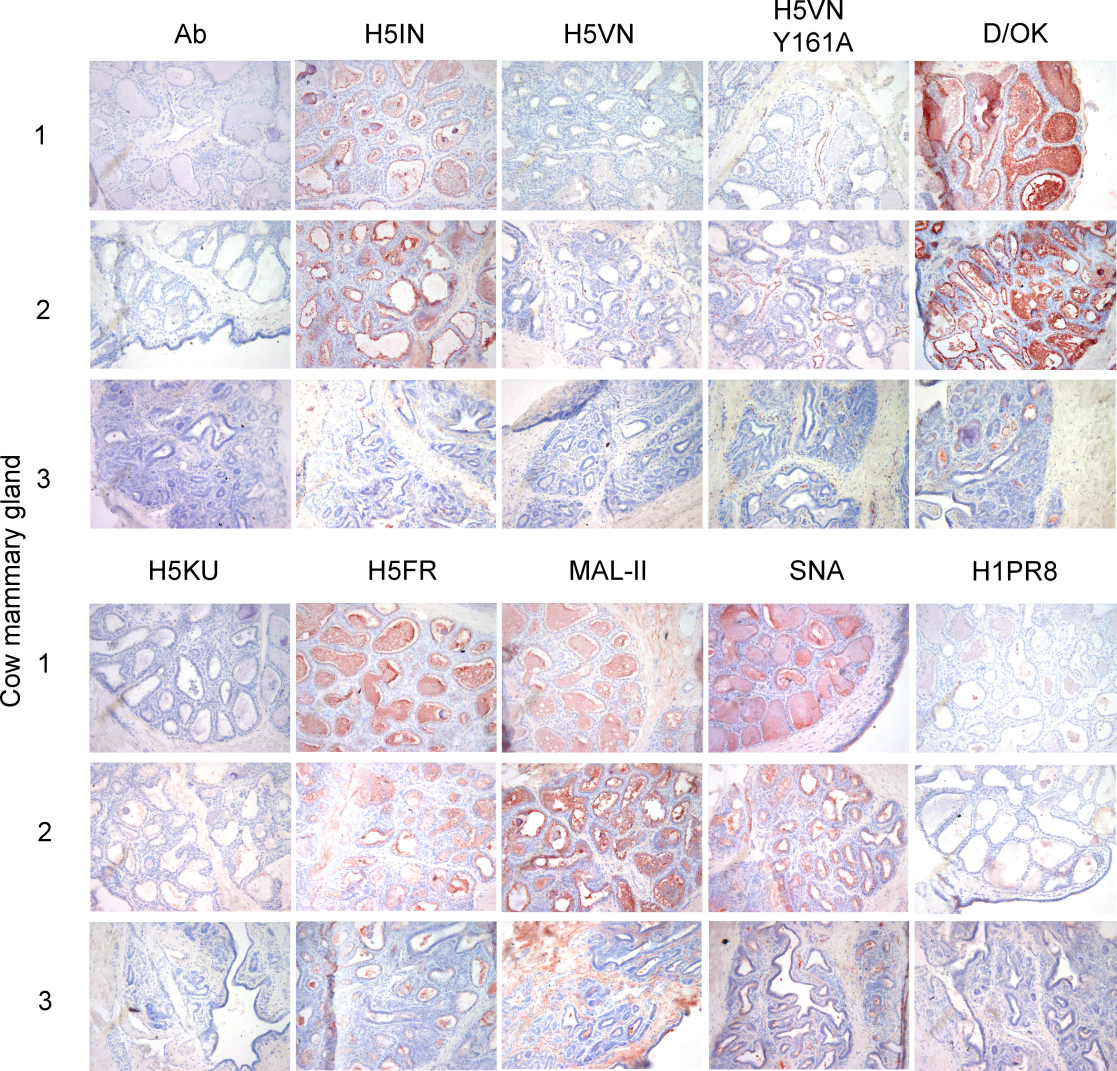
Immunohistochemical analysis of three cow mammary glands, stained with a small library of viral and plant lectins. The binding to mammary glands of three different cows was investigated for different IAV H5 proteins and a human H1 protein, as well as an IDV HEF and plant lectins MAL-II and SNA. 3-amino-9-ethylcarbazole staining was used to visualize tissue binding. Magnification is 20×.

We used FFPE tissues of the mammary glands of two lactating cows (1 and 2) and one non-lactating (3) cow and applied our library of plant-, IAV-, and IDV-derived glycan-binding proteins. In cows 1 and 2, the lumina were widened, containing proteinaceous material (Fig. 2). However, in cow 3, the minimal lumina observed did not contain proteinaceous fluid, and there was more fibrous tissue in the surrounding (38) (Fig. 2). No signal was observed when we applied our antibody mix as a negative control. The classical H5 proteins, H5IN and H5VN, showed variable binding in the mammary gland, with H5IN binding to cows 1 and 2 but not cow 3, exemplifying differences between lactating and non-lactating individuals (Fig. 2). In the case of the H5VN Y161A mutant, binding was observed in connective tissues and blood vessels (Fig. 2). The IDV hemagglutinin esterase fusion glycoprotein (HEF), on the other hand, bound throughout the entire mammary gland with high intensity, confirming the abundance of 9-O-acetylated Sia (Fig. 2).

We then applied a 2.3.4.4a (H5KU) and a 2.3.4.4b HA (H5FR) and observed that the H5KU did not engage Sias in the mammary gland, whereas the H5FR did (Fig. 2). Intense staining is observed within the mammary glands of all three cows for the latter. A similar observation is made for both plant lectins, MAL-II and SNA (Fig. 2). The distribution of α2,3 and α2,6 linked in the mammary gland from Holstein cattle has been recently studied using plant lectins (17). Even though lectin staining provides information on the display of sialic acids in a broad sense, it does not necessarily match with glycans that serve as IAV receptors (39). As an example, the H1 protein of a human H1N1 virus only displayed scattered binding in the mammary gland (Fig. 2), indicating that SNA might bind α2,6-linked Sias that are not specific receptors for human IAVs. For example, α2,6-linked Neu5Gc can be bound by SNA but is not a receptor of human influenza viruses (25). Furthermore, it is important to consider that the cows used for this study were not previously subjected to HPAIV H5N1 infection, and one of them was in non-lactating state, possibly presenting variable glycosylation patterns than those used in previous studies (17, 35).

**Fig 3:**
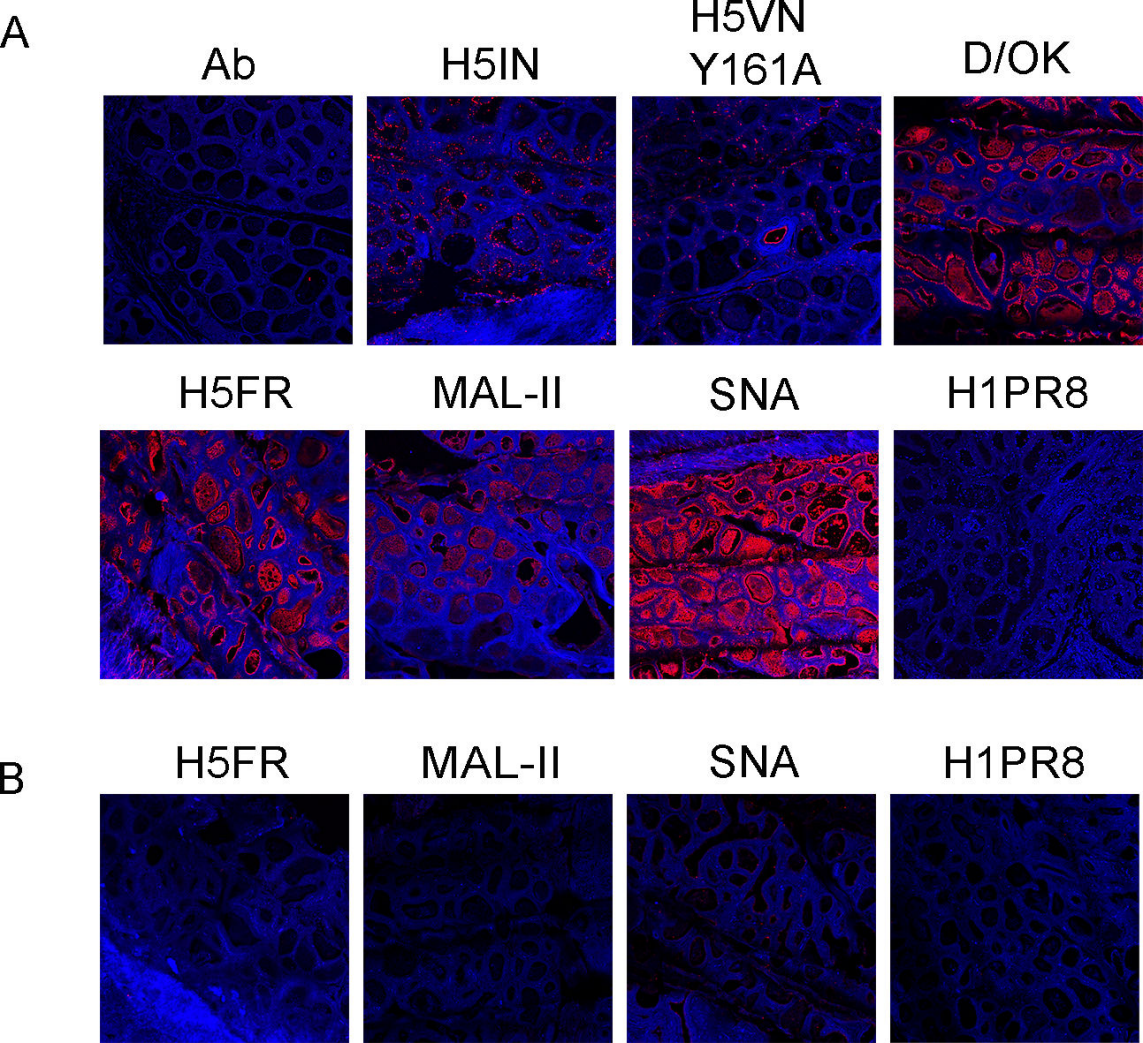
Immunofluorescent staining of a cow mammary gland, stained with a selected subset of viral and plant lectins. (**A**) To confirm our previous IHC results, binding was investigated for different classical H5IN and recent H5FR and H5VN Y161A mutants. A human H1 protein, an IDV HEF, and plant lectins MAL-II and SNA were used. (**B**) *Vibrio cholerae* neuraminidase treatment to assess sialic acid dependence. Alexa-555 dyes were used to visualize tissue binding. Magnification is 10× with 1.20× zoom.

Recently published work reported better sensitivity obtained with immunofluorescence (IF) compared to immunohistochemistry (IHC) (17). Given the quantitative limitations of IHC staining, we selected cow 1 to confirm our IHC now by immunofluorescent staining, with a representative subset of our lectin library (Fig. 3A). While H5IN showed binding, H5FR and FluD significantly outcompeted it in the mammary gland. The binding of H5VN Y161A was again more restricted to connective tissue and certain blood vessels, while H1PR8D hardly bound any mammary structure. For the plant lectins, both MAL-II and SNA showed binding, the latter being more intense and occurring in a broader range of structures. In contrast with Nelli et al. (17), our IF results are almost identical to IHC data, validating the quality of the assay.

We confirmed Sia dependency by treating the tissue sections with sialidase from *Vibrio cholerae* neuraminidase (VCNA), which completely removed binding from H5FR, H1PR8, and MAL-II (Fig. 3B). Although residual binding could be observed for SNA after VCNA treatment, the signal was significantly reduced. Conclusively, the mammary gland of cows displays receptors for 2.3.4.4b H5 proteins and further confirms the possibility of virus binding as the first step of active replication and transmission from this organ.

### The respiratory tract of cows hardly displays receptors for IAVs

In its wild waterfowl reservoir, IAVs are transmitted by the oral-fecal route, whereas in mammalian hosts, they are transmitted by the respiratory tract. Although all preliminary data points to a non-traditional transmission by milking practices, we did not want to exclude the respiratory tract of cows as a possible transmission route.

**Fig 4:**
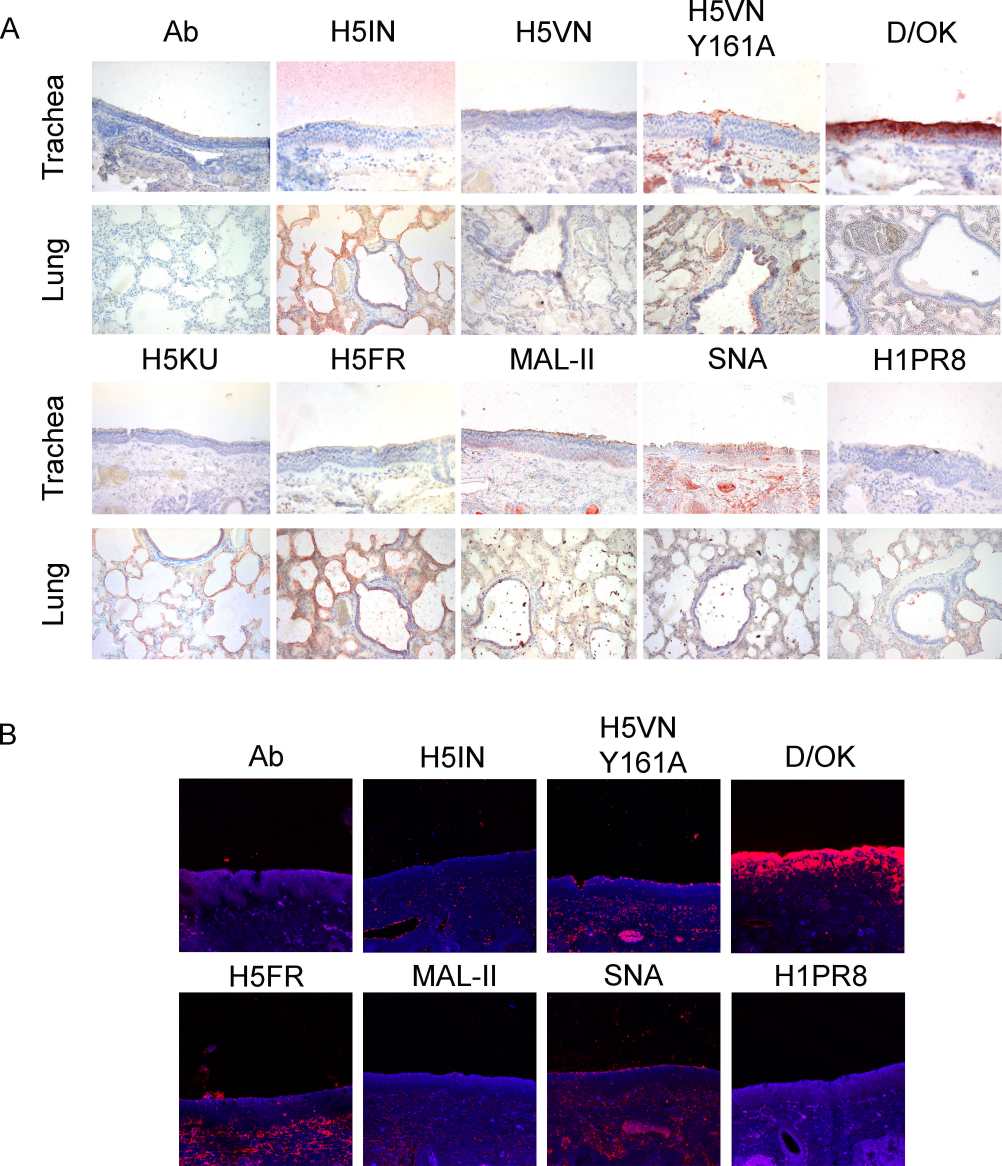
Immunohistochemical (**A**) and immunofluorescent (**B**) analyses of cow upper and lower respiratory tract tissues. The binding to upper (nasal or tracheal) and lower (lung) respiratory tract tissues of a cow was investigated for different IAV H5 proteins and a human H1 protein, as well as an IDV HEF and plant lectins MAL-II and SNA. 3-amino-9-ethylcarbazole staining and Alexa-555 dyes were used to visualize tissue binding. Magnification is 20× for IHC and 10× with 1.20× zoom for IF.

We used FFPE upper (nasal or trachea) and lower (lung) respiratory tract tissues from cows (*N* = 2) and applied our library of lectins. Data are shown for the same animal, as the other gave near identical results (Fig. 4A). The only H5 protein significantly binding to the cow upper respiratory tract was the H5VN Y161A mutant, which binds to 5-N-glycolyl Sia, which is not a receptor for circulating 2.3.4.4b H5N1 viruses. The lung tissues were bound by almost all HA proteins, including the human H1PR8, with variable intensity. Furthermore, the cow upper respiratory tract also abundantly displays 9-O-acetylated Sias, as indicated by the high signal intensity of D/OK. Both plant lectins bound to all tissues tested, contrasting the avian and human HAs in the tracheal and nasal tissues. This illustrates that plant-derived lectins, such as SNA and MAL-II, are poor predictors of IAV receptor distribution (39, 40). We confirmed our results with IF for a subset of proteins, obtaining similar results as for IHC (Fig. 4B).

The inability of our H5 proteins to engage in the upper respiratory tract of cows and the high abundance of IAV non-receptors such as 5-N-glycolyl and 9-O-acetyl most likely exclude this transmission route. Thus, receptors are available in the lungs, and lower respiratory tract infections are often not efficiently transmitted and cause severe disease (41). These findings align with a recently preprinted study by Halwe et al. (35), in which data suggest that several HPAIV H5N1 strains can replicate and successfully spread in the cow mammary gland but not in the respiratory tract.

### Glycan distribution in horse and pig’s respiratory tract illustrates differences in farm animals’ glycome

Contrary to cows, horses and pigs are classical hosts for IAV. Equine influenza is thought to have originated from avian IAVs, with the now-extinct highly pathogenic H7N7 and the currently circulating enzootic H3N8 (42–44). Given swine susceptibility to avian and human IAV infection, pigs are considered potential intermediate hosts for new reassortant viruses (45). Swine IAVs continue to circulate and cause limited enzootic outbreaks and sporadic pig-to-human transmission events (44).

**Fig 5:**
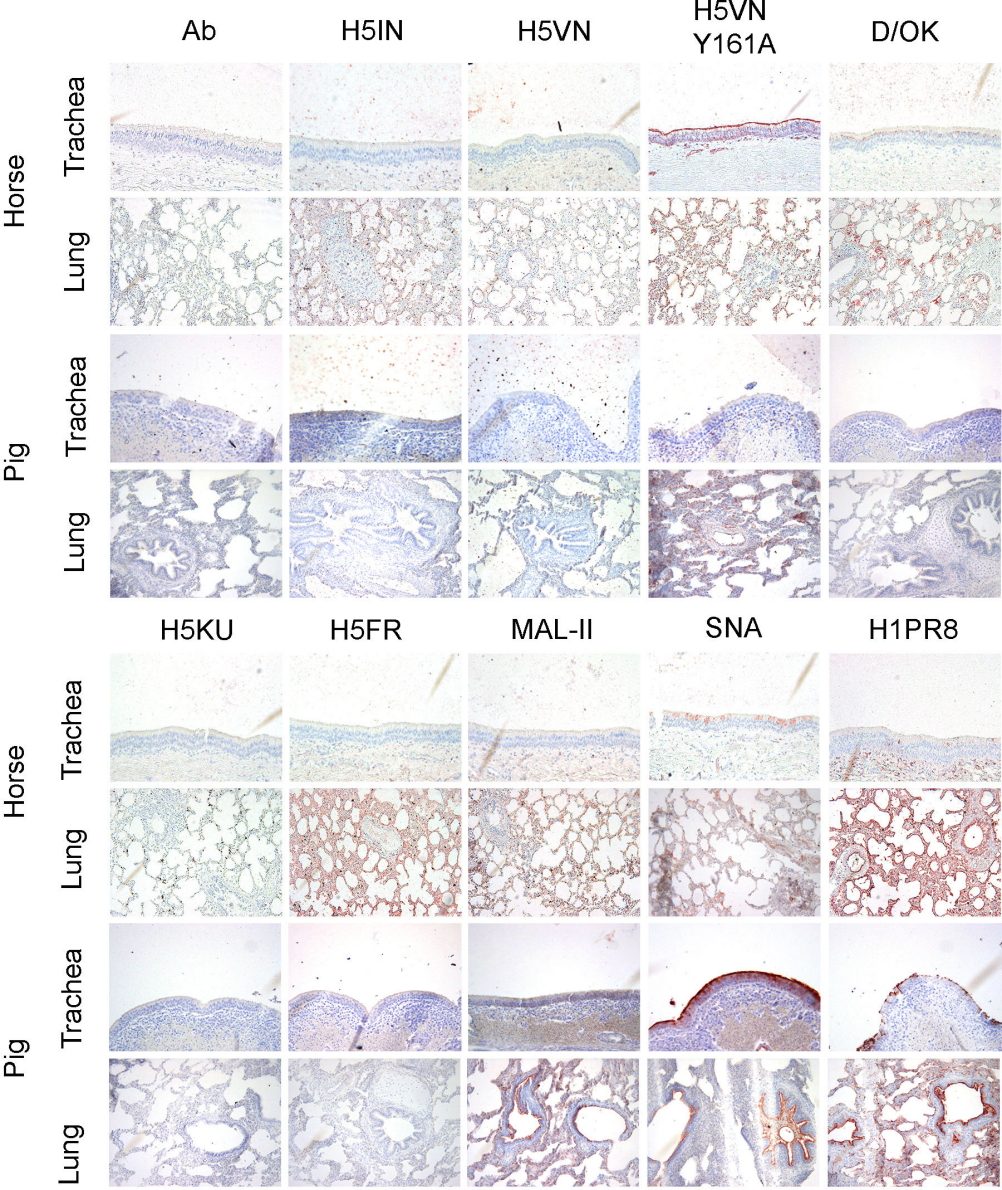
Immunohistochemical analysis of horse and pig upper and lower respiratory tract tissues. The binding to upper (nasal or tracheal) and lower (lung) respiratory tract tissues of a horse and pigs was investigated for different IAV H5 proteins and a human H1 protein, as well as an IDV HEF and plant lectins MAL-II and SNA. 3-amino-9-ethylcarbazole staining was used to visualize tissue binding. Magnification is 20×.

When we applied our library of lectins to the upper respiratory tract sections of horses and pigs (*N* = 2), we observed no binding for any of the wild-type H5 proteins used (Fig. 5). As expected, H5VN Y161A mutant strongly bound only to horse trachea due to its high content of α2,3-linked NeuGc (46). The upper respiratory tract of pigs predominantly expresses α2,6-linked NeuAc, which is not the preferred receptor of H5VN Y161A, hence the lack of binding (47). Plant lectin MAL-II staining showed the absence of α2,3-linked sialosides in the upper respiratory tract of these animals, whereas SNA revealed higher contents of α2,6-linked sialosides in pig compared to horse. Human H1PR8 showed the same pattern as SNA but with decreased intensity for both animals, again illustrating the broader binding profile of the plant lectin. Opposite to cows, D/OK stain demonstrated that the display of 9-O-acetylated Sias is significantly lower in horses, and not present in pigs, supported by previous MS data (31, 47) and the fact that pigs and horses are not hosts for IDV. These analyses revealed that 9-O-Ac-NeuAc could not be detected in pigs’ trachea and lungs, without excluding the possibility of other O-acetyl modifications present (47). Additionally, 4-O-acetyl is the most common modification of N-glycans in the upper respiratory tract of horses, specifically in α2,6-linked NeuAc receptors (31).

When moving deeper in the respiratory tract, horse lungs showed a similar pattern to cows, where almost all avian H5 proteins could engage receptors with variable intensity (Fig. 5). Contrasting to cows and horses, none of these proteins were able to bind the pig lung, except for the H5VN Y161A mutant. The content of α2,3 NeuGc in pigs’ lungs is minimal but still present (47). We observed a boost in human H1PR8 signal in horse lungs, while both MAL-II and SNA plant lectins stain showed a mixed distribution of α2,3- and α2,6-linked sialosides throughout the whole tissue. A similar pattern was observed in pig lungs, but the binding was restricted significantly to only bronchi and blood vessels. D/OK binding is now visible in some parts of the horse lung, while absent in pigs, the latter matching the observations in cows.

## DISCUSSION

Here, we demonstrate that the mammary gland of cows abundantly displays avian-type receptors for circulating H5 viruses. We also show that this organ lacks human-type receptors, which contradicts a previous study that only relied on plant lectins (16, 17). Furthermore, it has been reported that 2.3.4.4b H5s bound poorly terminally linked α2-6 sialic acid glycans printed on microarrays (48–50). Thus, we deem the adaptation of 2.3.4.4b H5N1 viruses to human-type receptor specificity during replication in the cow mammary gland unlikely. Nevertheless, in the reported human infections with cattle-derived strains, the virus is almost only isolated from eye swabs (39). Conjunctivitis in humans caused by AIV has been observed before and can be modeled in the ferret model (40, 41). After such ocular inoculation, the virus could further spread to the upper respiratory tract, in which H5N1 viruses might adapt to human-type receptors. Indeed, the first human case with respiratory tract infection has now been reported (51).

We focused our attention on the display of sialylated receptors for IAV HA proteins. We have previously shown that the receptor specificity of HA proteins is very similar to that of whole viruses, although with a lower degree of multivalency, leading to lower signals (24). However, we can easily observe binding in the cow’s mammary gland. Using multiple cows was vital as the signals in cow 3 are significantly lower, which probably presents natural variation. We would like to emphasize our use of an IDV HEF protein that stains the mammary gland tissues with high intensity. Cows are the reservoir for IDVs, and the abundant display of 9-O-acetylated Sias will hamper the binding of IAVs (31). Additionally, we would like to point out that the sole use of plant lectins to study the differential display of avian- and human-type receptors (36, 37) could lead to over-interpretations, as their binding profile is more promiscuous. As an example, using the human H1PR8 protein allowed us to determine that the presence of α2,6-linked sialosides does not necessarily mean that these are IAV receptors, as indicated by SNA staining.

Previous studies have focused on the glycan distribution in both the respiratory tract and the mammary gland of HPIAV H5N1-infected cattle in lactating state (17, 35). However, we also included a non-lactating cow in our study. Even though it is widely studied that glycosylation of milk proteins is altered during lactation stages in cows (52–54), it is still unknown if an HPAIV H5N1 infection or different lactating stages can influence the glycan profile in the mammary gland. Since the focus of the mentioned studies is based on α2,3- and α2,6-linked sialic acids (17, 35), we tried to dig deeper into a wider range of sialic acid modifications to highlight their importance in H5 hemagglutinin receptor-binding properties. Our observations match with the fact that binding in the mammary gland can support successful replication (35) and the abundance of α2,3 Sias in this organ (16, 17). Nevertheless, MS-based analyses are essential to further map the sialome.

Using direct IHC and IF binding of H5 proteins, we demonstrate that the upper respiratory tract of cows is devoid of receptors for IAV. We observe a similar pattern in horse and pig tracheas, which are classical IAV hosts, but no 2.3.4.4b H5N1 infections have been reported to this date. Thus, although the zoonotic transmission of 2.3.4.4b H5N1 viruses to ruminants is unprecedented, we would suggest that it does not pose an immediate pandemic risk, as there is no need to adapt to human-type receptors. However, the continuous and widespread circulation of these zoonotic viruses in primary livestock is a concern. While horses are hardly ever infected with avian IAVs, pigs are, and experimental infection of pigs with 2.3.4.4b viruses has shown that they are susceptible (43, 44), so the question is why we do not observe natural 2.3.4.4b H5N1 infections in pigs.

The main conclusion based on our results is that every large farm animal has a unique glycome. As we are currently experiencing, this allows circulating H5N1 viruses to adapt to new and possibly broader receptor specificities. While the upper respiratory tract of pigs is known to express predominantly α2,6-linked Neu5Ac and Neu5Gc with no 9-O-Ac modifications, horses come with higher expression of α2,3 Neu5Gc and the presence of 4-O-Ac-modified α2,6-linked Neu5Ac (31). In the case of cows, 5-N-glycolyl and 9-O-acetyls are abundantly displayed. These patterns change from the trachea to the lung, with consequences for infection. This is perhaps illustrated in a recent unofficial report that paints a picture of severe infections in cows, which might be related to lower respiratory tract infections, as ample receptors are available there (42).

The glycan display in the mammary gland indicates a possibility for a new viral replication and transmission route that could replace the classical oral transmission route in cows. We expect that our studies into the display of Sia modifications in ruminants will aid in our understanding of how different IAV viruses with distinct glycan specificities infect and transmit.

## MATERIALS AND METHODS

### Expression and purification of trimeric influenza A hemagglutinins

Recombinant trimeric IAV hemagglutinin ectodomain proteins (HAs) were cloned into the pCD5 expression vector (an example is Addgene plasmid #182546) in frame with a GCN4 trimerization motif (KQIEDKIEEIESKQKKIENEIARIKK), a super folder GFP or mOrange2 (55), and the Twin-Strep-tag (WSHPQFEKGGGSGGGSWSHPQFEK; IBA, Germany). The trimeric HAs were expressed in HEK293S GnTI(-) cells with polyethyleneimine I (PEI) in a 1:8 ratio (µg DNA:µg PEI) for the HAs as previously described. The transfection mix was replaced after 6 hours by 293 SFM II suspension medium (Invitrogen, 11686029), supplemented with sodium bicarbonate (3.7 g/L), primatone RL-UF (3.0 g/L, Kerry, NY, USA), glucose (2.0 g/L), glutaMAX (1%, Gibco), valproic acid (0.4 g/L), and DMSO (1.5%). According to the manufacturer’s instructions, culture supernatants were harvested 5 days post-transfection and purified with Sepharose Strep-Tactin beads (IBA Life Sciences, Germany).

### Protein histochemical tissue staining

Sections of archived formalin-fixed, paraffin-embedded cow, horse, and pig tissues were obtained from the Veterinary Pathology Diagnostic Centre, Faculty of Veterinary Medicine, Utrecht University, the Netherlands. The three cows used in this study were female individuals between 6 and 8 years old, admitted to the clinic due to previous cardiac and/or gastrointestinal pathologies. Cows, horses, and pigs were submitted for postmortem evaluation. No animals were euthanized for this study.

Protein histochemistry was performed as previously described (56, 57). In short, tissue sections of 5 µm were deparaffinized and rehydrated, after which antigens were retrieved by heating the slides in 10 mM sodium citrate (pH 6.0) for 10 min. Endogenous peroxidase was inactivated using 1% hydrogen peroxide in MeOH for 30 min at RT. Tissues were blocked at 4°C using 3% BSA (wt/vol) in PBS for at least 90 min. Subsequently, slides were stained for 90 min with 10 µg/mL solution of precomplexed proteins of interest. HAs were precomplexed with human α-strep-tag primary antibody and goat-α-human-HRP secondary antibody at a 4:2:1 molar ratio as previously described (12). For biotinylated plant lectins, we used streptavidin-HRP at a 4:1 molar ratio. We used 3-amino-9-ethylcarbazole (Sigma-Aldrich, Steinheim, Germany) to visualize protein binding. Tissue sections were then counterstained with hematoxylin and mounted with coverslips using AquaTex (Merck). Images were taken with an Olympus BX50 microscope and formatted for figure layout with Photoshop (Adobe) using a curves adjustment layer and increase (+50 points) in saturation. In the figures, representative images of at least two individual experiments are shown.

### Protein immunofluorescent tissue stain

Immunofluorescent staining was performed as previously described (27). Tissue sections of formalin-fixed, paraffin-embedded cow, horse, and pig used for IF were employed. These were deparaffinized and rehydrated, after which antigens were retrieved by heating the slides in 10 mM sodium citrate (pH 6.0) for 10 min. Endogenous peroxidase was inactivated using 1% hydrogen peroxide in MeOH for 30 min at RT. Tissues were blocked at 4°C using 3% BSA (wt/vol) in PBS for at least 90 min. Slides were then stained for 90 min with 10 µg/mL solution of precomplexed proteins of interest. HAs were precomplexed with human α-strep-tag primary antibody and goat-α-human-Alexa 555 secondary antibody at a 4:2:1 molar ratio as previously described (12). For biotinylated plant lectins, we used streptavidin-Alexa 555 at a 4:1 molar ratio. Nuclei were counterstained with 4′,6-diamidino-2-phenylindole, and samples were mounted with coverslips using FluorSave (Merck). Images were taken with a Leica DMi8 confocal microscope equipped with a 10× HC PL Apo CS2 objective (NA 0.40). Excitation was achieved with a Diode 405 or white light for excitation of Alexa555, with laser powers between 20% and 30%. LAS Application Suite X was used as well as ImageJ for image analysis.

## Data Availability

The authors declare that the data supporting the findings of this study are available within the paper and its supplementary information files.
